# Supplementary material for: Identification of Atypical Circulating Tumor Cells with Prognostic Value in Metastatic Breast Cancer Patients
Source: Cancers (Basel). 2022 Feb 13;14(4):932. doi: 10.3390/cancers14040932 (PMC8869799; doi:10.3390/cancers14040932)
Supplement: Supplementary file 1 [file cancers-14-00932-s001.zip › cancers-1543111-supplementary.pdf]

Supplementary Materials and Methods

Study Objectives and Design

PERMED-01 was a prospective unicentric clinical trial promoted by and conducted at the Paoli-Calmettes Institute (Marseille, France). Its primary objective was to evaluate the number of patients with locally advanced or metastatic cancer for whom identification of actionable genetic alterations (AGAs) in tumor samples using t-NGS and aCGH could lead to the delivery of a "matched therapy". Secondary objectives included the analysis of CTCs (for breast and digestive cancers), but also the assessment of feasibility, toxicity, and incidence on the clinical outcome of prospective molecular screening, the description of molecular alterations of advanced solid cancers and their relationship with the clinicopathological characteristics, their comparison with molecular alterations of the paired primary tumor if available, pan-genomic molecular analysis of metastatic samples with full exome sequencing and transcriptome analysis, analysis of circulating tumor DNA, and development of preclinical models for prediction/analysis of tumor response/resistance (xenografts, short-term culture, and organoids for breast cancer). Inclusion criteria were age ≥18 years, pathological diagnosis of solid cancer, locally advanced or metastatic stage progressive during at least one line of prior therapy and with an accessible lesion for biopsy, Eastern Cooperative Oncology Group (ECOG) Performance Status ≤2, affiliation to Social Insurance, and signed informed patient's consent for participation. Exclusion criteria were symptomatic or progressive leptomeningeal or brain metastases, bone or brain metastasis as sole metastatic site, pregnancy or breastfeeding, and person in an emergency situation or subject to a measure of legal protection or unable to express consent. The study, registered as ClinicalTrials.gov identifier NCT02342158, was approved by the French National Agency for Medicines and Health Products Safety, a national ethics committee (CPP Sud-Méditerranée), and our Institutional Review Board (Comité d'Orientation Stratégique, COS). It was conducted in accordance to the Good Clinical Practice guidelines of the International Conference on Harmonization. All patients gave their informed consent for inclusion, biopsy, and genomic analysis. Once enrolled, a new tumor biopsy or resection was proposed to the patient.

Statistical Analysis: Number of Inclusions

In order to have a sufficient number of patients with different cancers and with an identifiable AGA, we wanted to evaluate 300 patients enrolled over three years. Previous studies had reported a 35% technical failure rate. Thus, we initially planned to include 460 patients. On November 2017 after three years of inclusion and inclusion of the 460th patient, and in order to increase the numbers in certain patients' subpopulations for certain secondary objectives, we asked the French regulatory authorities to amend the protocol to allow enrollment of 100 additional patients over 1 year. The protocol was amended and the trial was reopened to inclusions on September 2018. On September 2019, after one year of inclusion and enrollment of 90 additional patients, the trial was closed. Thus, a total of 550 patients had been enrolled between November 2014 and September 2019, over less than 4 years of inclusion (November 2014 to November 2017, then September 2018 to September 2019).
In the present ancillary study on analysis of CTCs in patients with metastatic breast cancer, a total of 91 adult female patients had been enrolled after disease progression in our trial between January 2015 and December 2016 and were sampled for aCTC analysis. In addition, seven healthy adults lacking without any known pathology were recruited as controls.

Cell Lines

Cell lines were purchased from the ATCC® collection and cultured at 37°C and 5% CO_2 in recommended media. We used Hs 578T (Hs 578T ATCC ® HTB-126™), SK-BR-3 (ATCC® HTB-30™), SUM-190PT (BioIVT™) and MDA-MB-231 (ATCC® CRM-HTB-26™) breast cancer cell lines, Caco-2 (ATCC® HTB-37™) colon cancer cell line, and K-562 (ATCC® CCL-243™) myeloid cell line. Cell lines were regularly checked for mycoplasma contamination during their growth and discarded in case of positive results.

Setting up of 6-Color Immunofluorescence Using Cell Lines

Cell lines seeded on glass coverslips were fixed with 4% paraformaldehyde (Sigma Aldrich, Saint Quentin, France) for 5 minutes and permeabilized 5 minutes in TBS containing 0.2% Triton X-100 (Sigma Aldrich).

Each antibody used for the multicolor immunofluorescence (Table S7), was independently tested on positive and negative cells to establish the correct dilution and distinguish background from positive staining. EPCAM and pan-KRT antibodies, which are detected in the same channel in the multicolor combination, were also tested together. Multicolor immunofluorescence was conceived with the help of the application note from Zeiss (A ten-color spectral imaging strategy to reveal localization of gut immune cell subsets 2018 and [48]). The combination of secondary antibody fluorophores was chosen to allow spectral distinction (DL405, Sytox Blue, A488, DL549, A647, A680). All secondary antibodies were cross-adsorbed antibodies to minimize background and off-target signals. The correct separation of signals in the combination of primary and secondary antibody was confirmed with control staining on cell lines. The acquisition settings were maintained constant throughout the study and the apparatus performances are ensured by quality control of the imaging platform. The combination of antibodies is shown in Supplementary Table S7.

Evidences Suggesting that the Screencell®Cyto Device Do Not Generate CTM Artificially

To address potential artificial formation of clusters, 1,500 "single" cells from a cell line (SK-BR-3 or MDA-MB-231 cells, trypsinized, filtered with 70µm cell strainer and counted) were spiked into 3 ml of blood. This corresponds to the highest concentration of CTCs detected in our cohort. The blood was process within 4 hours with the ScreenCell®Cyto module (Sarcelles, France) and the filter was stained by May Grundwald Giemsa.

We then counted the number of clusters observed and documented their size. The result of 3 independent experiments is summarized in Supplementary Figure S6. Even though CTM can occasionally be observed after the filtration, the vast majority (> 92%) of cells are not observed within CTM.

CTM number and size distributions were compared to the data from 3 of our patients, displaying high number of CTM (#318 cells, #1443 cells and #1191 cells in total).

Most interestingly, in patients, the size of CTM was significantly bigger than CTM observed from spiked cells. Additionally, single CTCs (cluster's size <2 cells) were in minority compared to CTM, opposite to the situation observe in the test experiments. Altogether, this dampens the possibility that the filtration technic artificially induces the formation of cell clusters in general, and especially CTM above 3 cells, on the filter.

In addition, an indirect observation made from patients shows that high number of CTCs is not necessarily associated with the presence of CTM. For example, patient mBC#8 has a large total number of CTCs (60 CTCs/ml), similar to mBC#7 and m#BC9, but does not have many clusters compared to the other two patients. This precludes from artificial formation of CTM in samples with high number of CTCs.

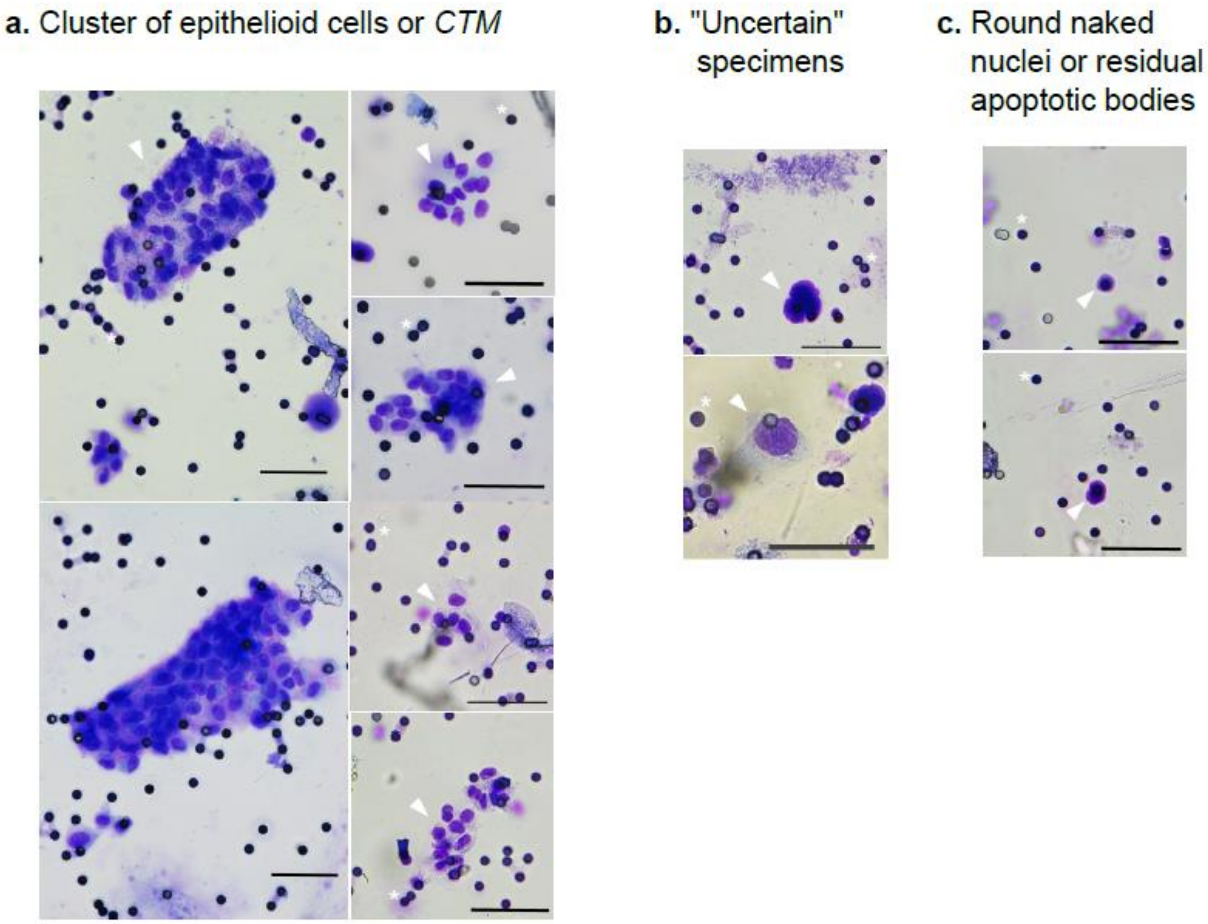

Figure S1. Pictures of atypical circulating cells observed in the blood of patients with mBC. Cytological staining with May-Grünwald solution of cells isolated from blood samples on a filter. Small black dots are filter pores (for clarity some examples are marked with a white asterisk). Scale represents 50µm. Cells of interest are marked with an arrow. The figure shows representative examples of: (**a**) Clusters of epithelioid cells of *CTM*; (**b**) “Uncertain” specimens, including cells with no clearly visible cytoplasm or without morphological malignant features. We observed samples with high frequency of dense and hyperchromatic nuclei (equal or greater than 20 µm) with irregular nuclear borders, but with no visible cytoplasm. These were naked nuclei or residual apoptotic bodies that we referred to as “uncertain” specimens, because the cellular origin cannot be addressed. These events were not analyzed in the present study. Complementary analysis is required to understand their origin: whether the pressure applied during the filtration blows apart the cytoplasm from the nucleus of fragile cells, or the cell is undergoing apoptosis in the bloodstream thereby displaying a retracted cytoplasm. Of note, they were not observed in control samples, and we cannot rule out a potential role in disease progression at this stage; (**c**) Round naked nuclei or residual apoptotic bodies. Scale represents 50µm. We also occasionally observed in patients and

control samples small (<20 µm) round naked nuclei or residual apoptotic bodies that were also not analyzed. Scale represents 50µm.

(**a**)

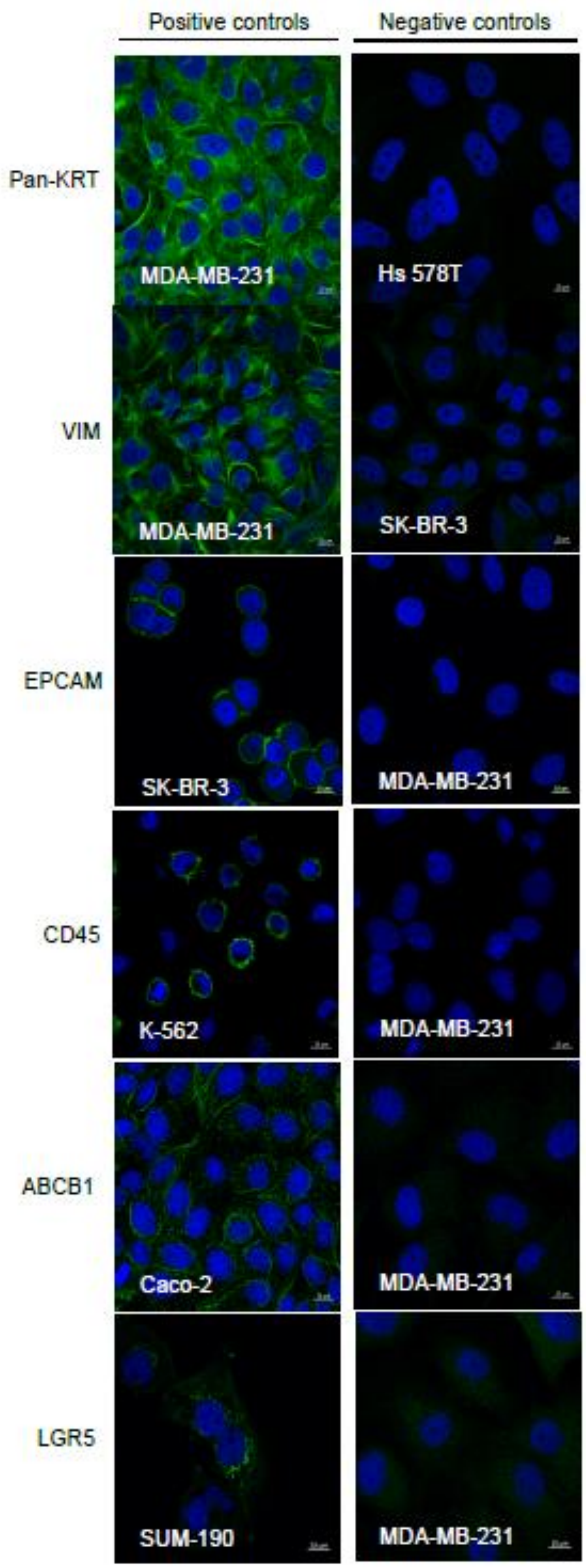
Positive controls
Negative controls
Pan-KRT
MDA-MB-231
Hs 578T
VIM
MDA-MB-231
SK-BR-3
EPCAM
SK-BR-3
MDA-MB-231
CD45
K-562
MDA-MB-231
ABCB1
Caco-2
MDA-MB-231
LGR5
SUM-190
MDA-MB-231

(b)

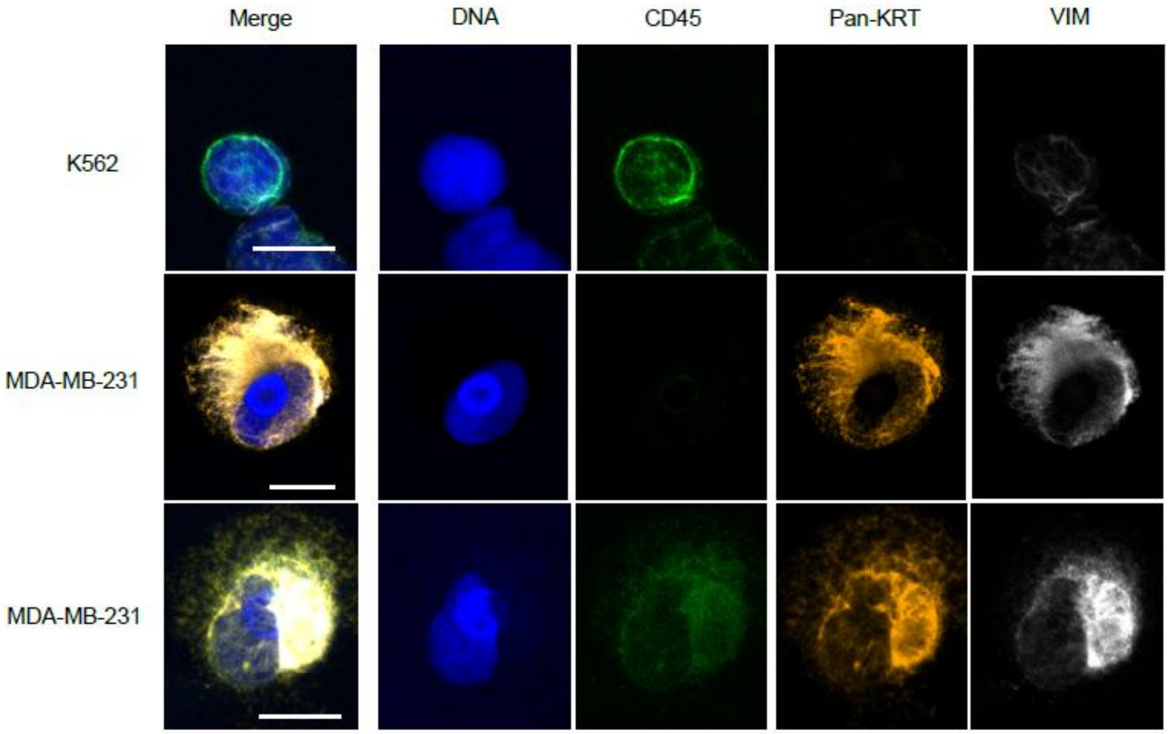

(c)

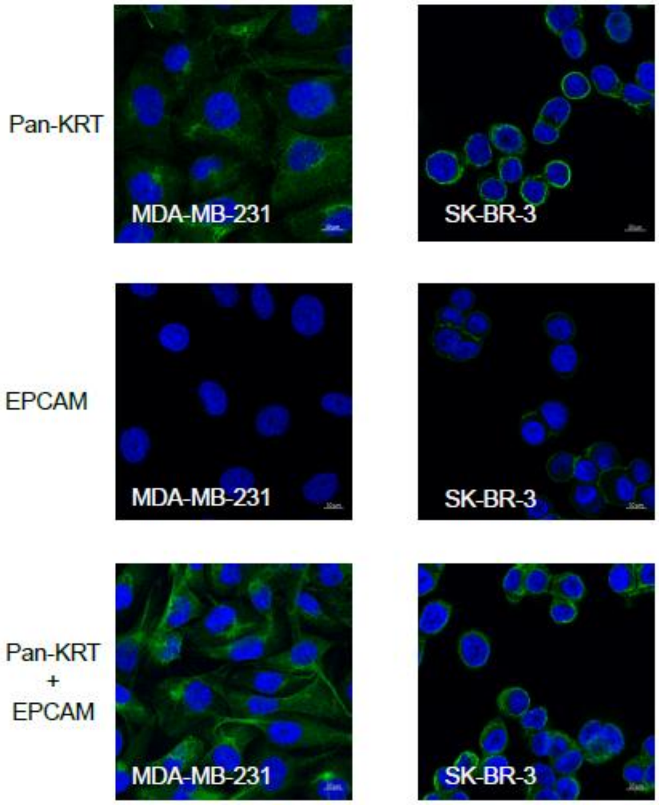

Figure S2. Antibody validations. (a). Antibodies specificity. Each antibody was tested on positive and negative control cells by immunofluorescence to assess their specificity and the signal/noise ratio. Pan-Cytokeratin showed a cytoplasmic staining in MDA-MB-231 (and SK-BR-3 not shown) cells. It was more or less filamentous depending on expression levels and cell spreading. Hs 578t cells were negative for cytokeratin staining. VIM antibody stained the cytoplasm of MDA-MB-231 cells but not SK-BR-3. Anti-EPCAM antibody stained the plasma membrane of SK-BR-3 cells but not MDA-MB-231. CD45 stained the plasma membrane of hematopoietic malignant cells, K-562, but not epithelial cells, MDA-MB-231. ABCB1 antibody was positive on Caco2 cells, with a predominant localization at the membrane (https://www.proteinatlas.org/ENSG00000085563-ABCB1) and negative on MDA-MB-231 cells. LGR5 was detected in a fraction of SUM190 cells, but not on MDA-MB-231 cells. LGR5 shows a dotted intracellular staining (Golgi apparatus and nucleoplasm) in agreement with data validated by the human protein atlas website (https://www.proteinatlas.org/ENSG00000139292-LGR5). LGR5 is not detected in MDA-MB231 cells. Of note, and as expected, markers staining's in cell lines were always stronger than what we could observe in the majority of cells isolated from patients in our study. **(b). Signal/noise ratio – Background.**

MDA-MB-231 cells, which are CD45- breast cancer cell line, grown in suspension (ultra-low adhesion plates) for two days were mixed with blood and filtered on a Screencell®Cyto device. Cells on the filter were immunostained as described in the material and methods to assess the immunolocalization of Pan-KRT and VIM in this condition. It showed that, despite a high expression of the two proteins, the filamentous aspect of Pan-KRT and VIM is less clear in cells in suspension compared to the same cells grown in 2D (**Supplementary Figure S2a**). It also highlights that, from the same cellular suspension, background intracellular CD45 staining can be detected in the cytoplasm of some epithelial cells. In this case, the CD45 labelling is diffuse and faint compared to the plasma membrane labelling observed on a CD45+ leukocyte. On the first line of images, we also added a staining with an anti-CD45 on hematopoietic malignant cells (K-562) to show a true signal against what is a background. This background "problem" can come from three causes. First, nonspecific background binding of antibodies (primary and secondary) can give a background signal. To limit this aspect, the saturation step included BSA, donkey and goat serum, and antibodies were titrated to be used at the right concentration for optimal signal to noise ratio. We cannot however control the state of each cells and quality of the filter that also affects the quality of the signal. The second cause is cellular auto-fluorescence that varies between cells. Auto-fluorescence is stronger and easier to detect on cells with bigger size and with large cytoplasm (in our case in g-*aCTCs* compared to leukocytes). The background is thus different depending on the cell type, cell size and cell shape. Third, auto-fluorescence is expected to vary with the metabolic activity of cells, and is enhanced in cells copping with severe stresses (auto-fluorescence is due to an increase in production of flavoproteins, which are involved in ROS detoxification). This explain why, within a sample, auto-fluorescence might differ from one cell to another [49]. This is rather intuitive to expect variation in auto-fluorescence of *aCTC* considering the stressful condition they are facing in the circulation. **(c). Antibodies combination validation**. Antibodies targeting different molecules corresponding to a given phenotype, here the epithelial phenotype, were simultaneously used and revealed in the same fluorescent canal. EPCAM and Pan-KRT antibodies were tested alone or mixed together to check that the combination of both antibodies allows them to conserve their specificity and to enhance the sensibility of detection of the desired phenotype. Scale bars: 10µm. The combination of all antibodies used on the filters was tested on cell lines to ensure its suitability. Cells were stained for CD45, Pan-KRT and VIM. Scale bars: 10µm.

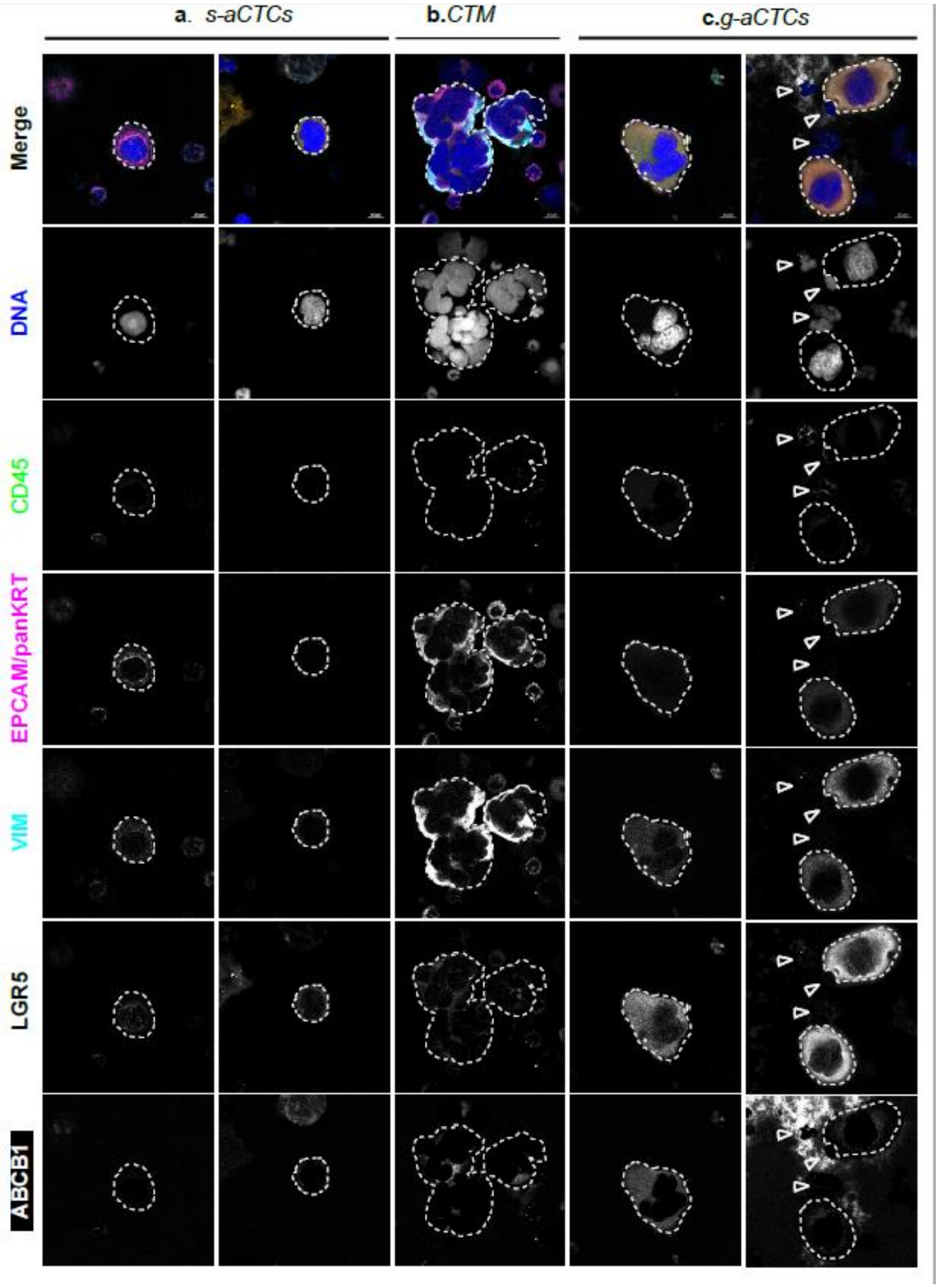

Figure S3. Examples of the immunofluorescence staining of atypical cells isolated on ScreenCell® filters. The blood of mBC patients were filtered and cells on the filter were immunostained. Blood cells are detected with the expression of CD45 antigen at the plasma membrane (empty arrowheads), whereas *aCTCs* (marked with dotted lines) are detected *via* the expression of epithelial markers (EPCAM and pan-KRT) and mesenchymal marker (VIM). Stem cell marker (LGR5) and drug resistance marker (ABCB1) expression are also assessed. Representative images of (**a**) *s-aCTCs:* CD45-, EPCAM/Pan-KRT-, VIM+, LGR5+, ABCB1+, and CD45-, EPCAM/Pan-KRT-, VIM+, Lgr5+, ABCB1-; (**b**) *CTM:* CD45-, EPCAM/Pan-KRT+, VIM+, LGR5+, ABCB1+, and; (**c**) *g-aCTCs*: CD45 diffuse, EPCAM/Pan-KRT+, VIM+, LGR5+,

ABCB1+, and CD45-, EPCAM/Pan-KRT+, VIM+, LGR5+, ABCB1-, are shown and indicated with dotted lines) Empty arrow heads point blood cells (CD45 positive staining the cell surface). Scale bar represents 10µm.

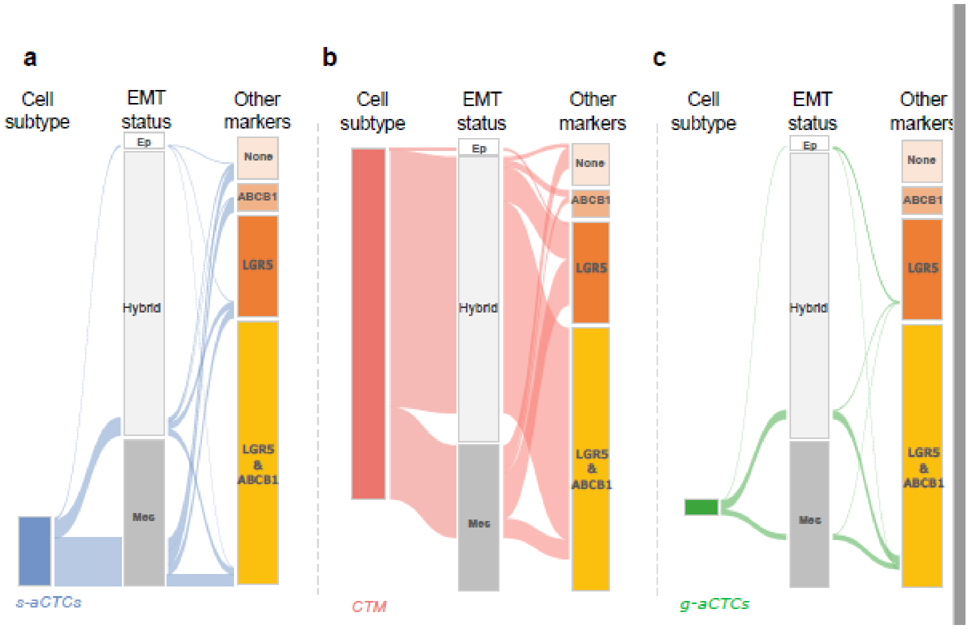

FigureS4. Alluvial plot representation of the combined expression of EMT, stem and drug resistance markers on aCTCs subsets individually (*CTM, g-aCTCs*, and *s-aCTCs*). The same data as in figure 3 are represented for each cell subset separately. Alluvial plot representation of the correlations between *aCTC* subsets and molecular markers. The graph shows for the three subsets of atypical cells separately (for *s-aCTCs* in **a**, for *CTM* in **b** and for *g-aCTCs* in **c**) the combined expression of EMT, LGR and ABCB1 markers. The height of the blocks represents the size of the population. The thickness of a stream represents the number of cells contained in blocks interconnected by the stream. EMT status: Ep=epithelial, Hybrid=epithelial + mesenchymal, Mes=mesenchymal; Other markers: ABCB1, LGR5, Mixed= ABCB1 + LGR5.

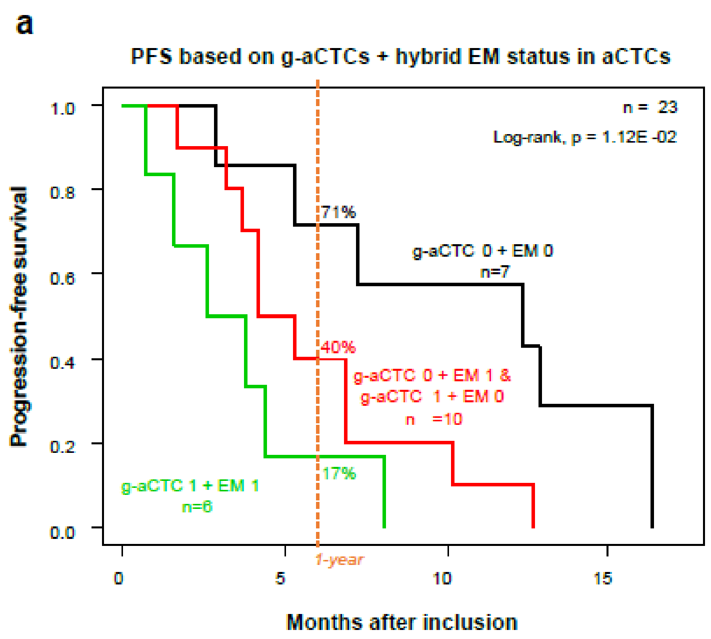

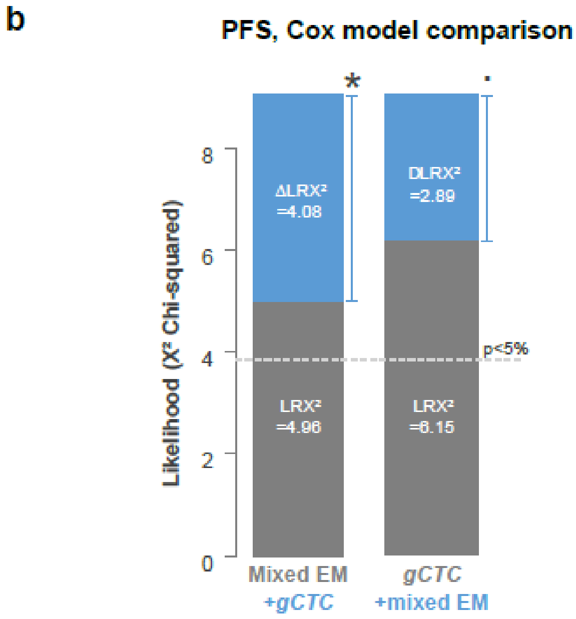

Figure S5. Kaplan-Meier curves of PFS of patients based on the positivity of *g-aCTCs* and intermediate EM status. (**a**) Survivals were calculated using the Kaplan-Meier method and were compared with the log-rank test to evaluate the prognostic value of combined *g-aCTC* and intermediate EM statuses. Presence of *g-aCTC* is marked as 1 vs absence as 0. Presence of an intermediate EM status in all aCTCs' subsets is represented as 1 value, absence as 0; (**b**) Prognostic complementarity: the values are given for prognostic information of each variable colored in grey (Mixed EM and *g-aCTC*) on its own (LR-χ2) and

when added to the other variable colored in blue (ΔLR-χ2). * indicates p≤0.05 and indicates trend for significance with p≤0.10.

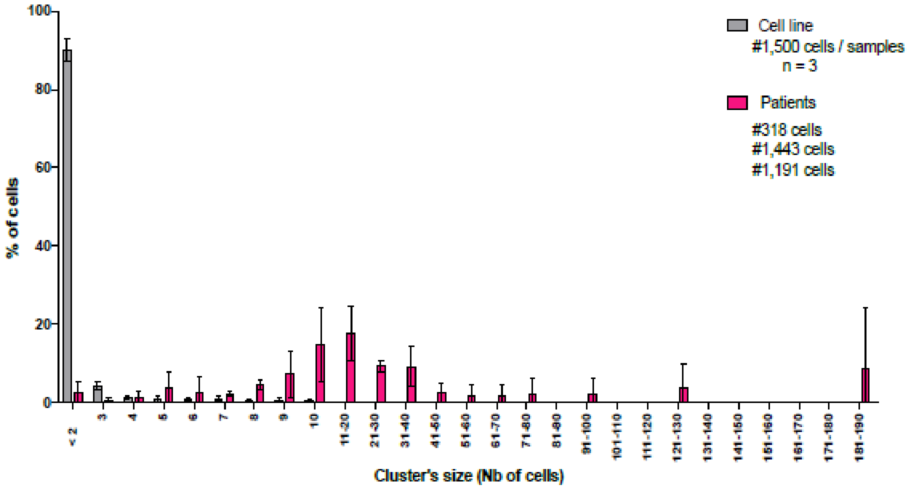

Figure S6. Clusters verification. To address potential artificial formation of clusters during the filtration process 1,500 "single" cells (which corresponds to the highest concentration of *a-CTCs* observed in patient) from a cell line SK-BR-3 or MDA-MD-231 (trypsinized and counted) were mixed into 3 ml of blood. The blood was then process as usually within 4 hours with the ScreenCell®Cyto module and stained with May Grundwald Giemsa to count clusters. The results are compared with cluster occurrence in patient samples containing an equivalent number of *a-CTCs* (318, 1,191 and 1,443 *a-CTCs* in 3mls of blood).

Supplementary Tables

Table S1. Summary of systemic therapies received before and after inclusion.

	Therapy	Before inclusion	After inclusion
Number of previous lines of systemic, median (range)	chemotherapy	3 (1-7)	1 (0-6)
	hormone therapy	1 (1-5)	0 (0-2)
	immune therapy	0 (0-1)	0 (0-1)
	targeted therapy	1 (0-5)	1 (0-2)
Number of patients pre-treated with systemic, N (%)	chemotherapy	85 (93%)	84 (92%)
	hormone therapy	55 (60%)	26 (29%)
	immune therapy	1 (1%)	3 (3%)
	targeted therapy	59 (65%)	42 (54%)

Table S2. Distribution of aCTC subsets in the population of mBC patients.

	**All atypical circulating cells (s-a*CTCs, CTM, g-aCTCs*)**	***s-aCTC***	***CTM***	***g-aCTC***
Median value#	8.33	2	1.33	0
Min - Max values#	0 - 481.6	0 - 51	0 - 479.6	0 - 10.3
Mean [SD] value#	27.17 [+/- 71.8]	4.0 [+/- 6.7]	21.9 [+/- 71.1]	1.2 [+/- 2.1]
Nb of subjects with ≥ 1 event / filter	87/91	72/91	47/91	33/91
% of subjects with ≥ 1 event / filter	96%	79%	52%	36%
Nb of subjects defined as positive¤	41	49	22	42
% of subjects defined as positive¤	45%	54%	24%	46%

s-aCTCs: Single epithelioid cells; CTM: cluster of cells; g-aCTCs: Giant epithelioid cells. Nb: Number; SD: Standard deviation; Min: minimal; Max: maximal. Numbers of CTCs are per mL. **#:** values per ml of blood per patient; **¤:** using cut-off specific for each cell type.

Table S3. Correlations between *aCTCs* and *aCTC* subsets and clinicopathological variables. #, variables tested as continuous values.

	**All atypical circulating cells**			***s-aCTCs***			***CTM***			***g-aCTCs***		
	N	Odds ratio [95%CI]	p-value	N	Odds ratio [95%CI]	p-value	N	Odds ratio [95%CI]	p-value	N	Odds ratio [95%CI]	p-value
Age at inclusion#, years	91	1.00 [0.97-1.03]	0.83	91	0.98 [0.95-1.01]	0.219	91	0.99 [0.96-1.02]	0.556	91	1.00 [0.97-1.04]	0.823
Metastasis to diagnosis interval#, years	91	0.99 [0.93-1.06]	0.819	91	1.04 [0.97-1.11]	0.372	91	0.92 [0.86-0.98]	**0.0451**	91	1.07 [0.99-1.16]	0.089
Pathological type primary tumor, lobular vs. ductal	81	1.34 [0.33-5.43]	0.728	81	0.83 [0.21-3.35]	0.825	81	2.00 [0.45-8.98]	0.448	81	3.77 [0.86-16.55]	0.14
Pathological grade primary, 2 vs. 1	74	4.5e-08 [0e+00-Inf]	0.992	74	0.28 [0.04-1.95]	0.282	74	0.76 [0.15-3.78]	0.775	74	0.59 [0.12-2.94]	0.588
3 vs. 1	74	2.5e-08 [0e+00-Inf]	0.992	74	0.24 [0.035-1.61]	0.216	74	0.70 [0.14-3.47]	0.717	74	0.21 [0.042-1.09]	0.12
Molecular subtype of primary, HER2+ vs. HR+/HER2-	90	0.43 [0.14-1.30]	0.21	90	0.98 [0.32-3.08]	0.982	90	1.06 [0.35-3.19]	0.93	90	0.35 [0.10-1.16]	0.148
TN vs. HR+/HER2-	90	0.73 [0.33-1.61]	0.512	90	0.30 [0.13-0.66]	**0.0129**	90	2.42 [1.09-5.36]	**0.0339**	90	0.65 [0.30-1.42]	0.364
Molecular subtype of metastasis, HER2+ vs. HR+/HER2-	87	0.68 [0.22-2.14]	0.578	87	0.47 [0.15-1.48]	0.281	87	1.68 [0.54-5.26]	0.454	87	0.46 [0.14-1.47]	0.271
TN vs. HR+/HER2-	87	0.76 [0.35-1.66]	0.57	87	0.42 [0.19-0.91]	**0.0322**	87	1.89 [0.88-4.07]	0.17	87	0.59 [0.28-1.27]	0.257
Bone metastasis, yes vs. no	91	1.72 [0.82-3.60]	0.226	91	1.87 [0.90-3.89]	0.157	91	1.34 [0.65-2.77]	0.503	91	0.65 [0.31-1.34]	0.327
Liver metastasis, yes vs. no	91	2.60 [1.25-5.38]	**0.0313**	91	2.07 [1.02-4.21]	**0.04555**	91	1.76 [0.87-3.57]	0.184	91	1.00 [0.50-2.01]	1
Lung-Pleural metastasis, yes vs. no	91	1.37 [0.67-2.79]	0.466	91	1.49 [0.74-2.98]	0.349	91	1.49 [0.74-2.97]	0.348	91	0.96 [0.48-1.92]	0.923
Brain-meningeal metastasis, yes vs. no	91	2.75 [0.71-10.63]	0.219	91	2.17 [0.66-7.14]	0.286	91	1.46 [0.48-4.50]	0.577	91	3.07 [0.93-10.12]	0.123
Lymph node metastasis, yes vs. no	91	0.81 [0.39-1.69]	0.632	91	0.72 [0.35-1.49]	0.463	91	0.76 [0.37-1.56]	0.533	91	0.78 [0.38-1.60]	0.57
Skin metastasis, yes vs. no	91	0.73 [0.31-1.76]	0.561	91	0.35 [0.14-0.87]	**0.0286**	91	0.92 [0.39-2.19]	0.876	91	0.51 [0.21-1.27]	0.228
Peritoneum metastasis, yes vs. no	91	2.75 [0.71-10.63]	0.219	91	1.33 [0.43-4.08]	0.68	91	0.59 [0.19-1.81]	0.438	91	5.53 [1.43-21.37]	**0.0375**
Other metastasis sites, yes vs. no	91	0.91 [0.41-2.02]	0.853	91	0.46 [0.21-1.01]	0.106	91	0.82 [0.38-1.77]	0.668	91	0.71 [0.32-1.55]	0.47
Number of metastatic sites at inclusion#, N	91	1.16 [0.92-1.47]	0.302	91	1.04 [0.83-1.30]	0.77	91	1.08 [0.87-1.36]	0.556	91	0.98 [0.78-1.22]	0.875

Number of previous lines of systemic therapy at inclusion#, N	91	1.13 [0.97-1.31]	0.19	91	1.04 [0.90-1.20]	0.667	91	1.09 [0.94-1.26]	0.329	91	1.17 [0.91-6.51]	0.0838
All atypical cells, positive vs. negative	---	---	---	91	4.61 [2.15-9.87]	**0.000973**	91	13.21 [5.43-32.14]	**0.0000018**	91	2.71 [1.29-5.70]	**0.0279**
s-aCTC, positive vs. negative	91	4.61 [2.15-9.87]	**0.000973**	---	---	---	91	0.79 [0.40-1.59]	0.582	91	3.95 [1.88-8.28]	**0.00229**
CTM, positive vs. negative	91	13.21 [5.43-32.14]	**0.0000018**	91	0.79 [0.40-1.59]	0.582	---	---	---	91	0.74 [0.37-1.48]	0.477
g-aCTC, positive vs. negative	91	2.71 [1.29-5.70]	**0.0279**	91	3.95 [1.88-8.28]	**0.00229**	91	0.74 [0.37-1.48]	0.477	---	---	---

Table S4. Molecular markers expressed by *aCTCs.*

			**Percentage of *aCTCs* positive for:**						
			**Leukocyte marker**	**EMT status**				**Stem-like and drug resistance markers**	
**Subsets**	**Total nb of cells analyzed**	**Nucleus size, mean (SD)**	**CD45 (%)**	**Epithelial markers (%)**	**Epithelial + mesenchymal markers (%)**	**Mesenchymal markers (%)**	**None of the EMT markers (%)**	**LGR5 marker (%)**	**ABCB1 marker (%)**
s-aCTC	336	20.8 (+/-3.8)	0	2.6	27.5	69.9	0	52.1	49
CTM	1742 (164 cl)	16.3 (+/-3.6)	0	0.6	82.5	16.4	0.5	89.4	69.4
g-aCTC	74	29.8 (+/-8.3)	35 (faint & cytoplasmic)	0	63.6	27.4	9	98.9	89.3

Table S5. Univariate analysis for PFS according to molecular and cytological profiles.

PFS		Univariate		
		N	HR [95%CI]	p-value
Epithelial	positive vs. negative	23	0.69 [0.16-3.02]	0.623
Mixed epithelial-mesenchymal	positive vs. negative	23	2.87 [1.08-7.68]	**0.0353**
Mesenchymal	positive vs. negative	23	0.73 [0.30-1.75]	0.483
All atypical circulating cells (aCTCs)	positive vs. negative	23	1.72 [0.72-4.11]	0.218
s-aCTC	positive vs. negative	23	1.67 [0.68-4.11]	0.263
CTM	positive vs. negative	23	0.93 [0.37-2.35]	0.882
g-aCTC	positive vs. negative	23	3.41 [1.20-9.69]	**0.0212**

Table S6. Summary of the cytomorphological criteria used to define each *aCTC* subset.

Characteristics	*s-aCTC*	*CTM*	*g-aCTC*	/!\ Might be observed on
Irregularity of nuclear borders	Yes	Yes	Yes	Endothelial circulating cells found in microemboli (tEC-CTM), megakaryocytes
Dense hyperchromatic nucleus	Yes	Yes	Yes	megakaryocytes
Morphology variants	Round	Cluster of cells	Amorphous, oblong, spindle-shaped, round, and tadpole-shaped	
Anisonucleosis	-	> 0.5	-	
Number of cells	1	≥ 3	1	Endothelial circulating cells found in microemboli (tEC-CTM)
Multilobular or with separated polymorphic nuclei	No	No	Yes	megakaryocytes
Cell size	Variable	usually < 50µm	≥ 50	megakaryocytes
Nucleus size	≈ 20µm (+/-4 µm in the literrature)	< 20µm	≥ 20µm	megakaryocytes (enlarged nuclei)
Nucleocytoplasmic (N/C) ratio	High (N/C > 0.75)	High to Medium	Low (0.5 > N/C)	megakaryocytes (Low)

Table S7. Antibodies used for immunofluorescence.

Primary antibodies

Antibody name	Type	Supplier	Reference	Remarks
LGR5	rabbit polyclonal	Abgent	AP2745D	
Pan-cytokeratin C-11	mouse IgG1	Abcam	ab7753	Peptides to KRT4, KRT5, KRT6, KRT8, KRT10, KRT13, KRT18
EPCAM	mouse IgG1	Invitrogen	MA5-12153	
VIMENTIN	chicken poyclonal	R&D system	NB300-223	
ABCB1	mouse IgG2b	Becton Dickinson	557001	
CD45-AF488	mouse IgG1	Biolegend	304017	Antibody la-belled with alexa fluor 488

Secondary antibodies

Antibody name	Fluorophore	Supplier	Reference
Goat anti-mouse IgG2b	DL405	Jackson ImmunoResearch	115-475-207
Donkey anti-rabbit	DL549	Jackson ImmunoResearch	711-166-152
Goat anti-mouse IgG1	AL647	Jackson ImmunoResearch	115-605-205
Gonkey anti-chicken	A680	Jackson ImmunoResearch	703-625-155
